# Cross-Kingdom Regulation of Putative miRNAs Derived from Happy Tree in Cancer Pathway: A Systems Biology Approach

**DOI:** 10.3390/ijms18061191

**Published:** 2017-06-03

**Authors:** Dinesh Kumar, Swapnil Kumar, Garima Ayachit, Shivarudrappa B. Bhairappanavar, Afzal Ansari, Priyanka Sharma, Subhash Soni, Jayashankar Das

**Affiliations:** Gujarat Institute of Bioinformatics, Gujarat State Biotechnology Mission, Department of Science & Technology, Government of Gujarat, Gandhinagar 382011, India; dinesh.vascsc@gmail.com (D.K.); swapnilkr.bi@gmail.com (S.K.); garima_ayachit@yahoo.co.in (G.A.); rudra8282@gmail.com (S.B.B.); md.afzal294@gmail.com (A.A.); mailsharma.p@gmail.com (P.S.); mdbtm@gujarat.gov.in (S.S.)

**Keywords:** miRNA, *Camptotheca acuminata*, cancer, cross-kingdom regulation, protein-protein interaction network

## Abstract

MicroRNAs (miRNAs) are well-known key regulators of gene expression primarily at the post-transcriptional level. Plant-derived miRNAs may pass through the gastrointestinal tract, entering into the body fluid and regulate the expression of endogenous mRNAs. *Camptotheca acuminata*, a highly important medicinal plant known for its anti-cancer potential was selected to investigate cross-kingdom regulatory mechanism and involvement of miRNAs derived from this plant in cancer-associated pathways through in silico systems biology approach. In this study, total 33 highly stable putative novel miRNAs were predicted from the publically available 53,294 ESTs of *C. acuminata*, out of which 14 miRNAs were found to be regulating 152 target genes in human. Functional enrichment, gene-disease associations and network analysis of these target genes were carried out and the results revealed their association with prominent types of cancers like breast cancer, leukemia and lung cancer. Pathways like focal adhesion, regulation of lipolysis in adipocytes and mTOR signaling pathways were found significantly associated with the target genes. The regulatory network analysis showed the association of some important hub proteins like GSK3B, NUMB, PEG3, ITGA2 and DLG2 with cancer-associated pathways. Based on the analysis results, it can be suggested that the ingestion of the *C. acuminata* miRNAs may have a functional impact on tumorigenesis in a cross-kingdom way and may affect the physiological condition at genetic level. Thus, the predicted miRNAs seem to hold potentially significant role in cancer pathway regulation and therefore, may be further validated using in vivo experiments for a better insight into their mechanism of epigenetic action of miRNA.

## 1. Introduction

Non-coding RNAs (ncRNA) are evolving as key players in regulating gene expression of constitutive as well as signaling processes inside cells. MicroRNAs (miRNAs), discovered for the first time in *C. elegans* is a class of ncRNAs, 18 to 24 nucleotide long, act as a molecular switch in post-transcriptional regulation [1]. Plant miRNAs have remained conserved during the course of evolution, owing to their significant role in several biological functions such as plant tissue development [2], floral patterns [3], response to abiotic and biotic stress [4] and signaling [5]. In animal kingdom, miRNAs are known to regulate different cellular processes such as cell proliferation [6], cell death [7], fat metabolism [8], neuronal patterning [9], stem cell differentiation [10] and immune response [11].

It has been well studied that plant derived miRNAs may pass through gastrointestinal (GI) tract releasing biomolecules like amino acids, fatty acids and miRNAs after digestion which get absorbed by human cells. The epithelial cell lining of intestine may absorb these exogenous plant miRNAs packaged into microvesicles (MVs) along with RNA induced silencing complex (RISC) components subsequently reaching the destination through circulatory system i.e., the recipient cells by escaping other barriers of digestive system and RNase digestion. miRNAs get released and participate in regulation of the target gene. It has been demonstrated that fluorescently labelled miRNAs packaged in MVs can get delivered to the recipient cells and can regulate the expression of target genes. After reaching the recipient cells, the plant miRNAs follow several strategies to ensure their regulatory functions which can be understood by the fact that MVs not only package miRNAs but also RISC components to guarantee the active status of the packaged miRNAs [12,13]. The RNA transporter protein may also be a means of transportation for the exogenous miRNAs across the mammalian intestinal tract, which are present on the cell surface [14].

Plant miRNAs are known to be methylated on the 2-hydroxyl group of 3-terminal nucleotide, which result in the inhibition of 3′-uridylation and subsequent digestion by 3–5 exonuclease [15]. The plant-derived miRNAs have low degradation rate than that of synthetic forms [16]. It suggests that the methylation of plant miRNAs may contribute to their stability. Further, it has been verified in several experimental studies on *Brassica oleracea*, *Oryza sativa* and *Lonicera japonica* (honeysuckle) using northern blot and qRT-PCR methods that the detected miRNAs in animals are authentic plant miRNAs [17,18,19].

Increasing experimental findings and evidence suggests that cancer stem cells (CSC) associated miRNAs could play important roles in suppression and inhibition of different types of tumor development and progression [20]. For example, in mammals, miR-7, miR-129-5p, miR-490-3p and miR-204 and miR-211 have been proven to act as tumor suppressors [21], inhibiting the progression and proliferation of hepatocellular carcinoma [22], pulmonary and intestinal carcinoma [23] and breast cancer [24].

In addition to mammalian miRNAs, plant derived miRNAs are also known to regulate target genes of mammals in a cross kingdom manner as evident from several recent studies. One of the first evidence of this cross-kingdom regulation in human/mouse as miR-168a from *Oryza sativa* could bind to the human/mouse LDLRAP1 (Low-density lipoprotein receptor adapter protein 1) mRNA by inhibiting its expression in liver and consequently decreasing LDL removal from mouse plasma [16]. Later on, miR-2911 from honeysuckle showed that it can target influenza A virus (IAV), especially H1N1, H5N1, and H7N9 in mice [25]. Further, an in vitro study showed that miR160 and miR2673 from *Brassica oleracea* can regulate the expression of human lung cancer related genes and proteins [26]. Another recent investigation has indicated that oral administration of plant miR159 mimic suppress xenograft breast tumor growth in mice and was capable of inhibiting proliferation by targeting TCF7 that encodes a Wnt signaling transcription factor, leading to a decrease in MYC protein levels. These results proved for the first time that a plant miRNA can inhibit cancer growth in mammals [27].

Medicinal plants offer enormous benefits and unexplored genetic diversity with unique endemic characteristics. Natural products derived from the medicinal plants often possess biological activities that may be valuable in the development of new therapeutic agents for treatment of variety of diseases [28,29,30,31]. Many evidences of intake and bioavailability of plant derived miRNAs in humans and animals have been explored for their immuno-modulating capacity [32]. These small RNAs can control the gene expression and may provide an effective, noninvasive, and inexpensive therapy for many human diseases [33]. Given the immense possibility, the therapeutic potential of miRNAs and their mechanism of cross-kingdom gene regulation need to be further explored and verified. It is expected that targeting endogenous mRNAs by plant-derived miRNAs may prove to be a novel approach in cancer treatment by combining traditional therapeutics with medicinal products to increase overall response.

*Camptotheca acuminata* Decne. (Nyssaceae) is a deciduous tree having anti-cancer properties and commonly known as Happy Tree. The extracts of fruits and leaves of *C. acuminata* in aqueous form have been used from a long time in the Indian and Chinese systems of traditional medicine for treating different types of cancers [34,35]. Camptothecin (CPT), a terpenoid indole alkaloid (TIA) derived from this plant is known to be the major traditional drug used as potent chemotherapeutic agent [36,37,38]. The compound and its derivatives are known to inhibit the nuclear enzyme DNA topoisomerase I and was first isolated from the bark and stem of *C. acuminata* in 1966 [39]. Its two derivatives viz. Irinotecan (9-[(dimethylamino)methyl]-10-hydroxy-camptothecin) and Topotecan (7-ethyl-10-[4-(1-piperidino)-1-piperidino] carbonyloxycamptothecin), have been approved by Food and Drug Administration (FDA) for treating colorectal and ovarian cancer [30,40,41,42]. Aqueous extracts of fruit of *C. acuminata* (AE-CA) is more efficient than CPT itself in terms of tumor suppression efficiency and cytotoxicity as reported in a study on the endometrial carcinoma cell lines of human (HEC-1A, HEC-1B, and KLE) [43].

As evident, it has been proven that one miRNA can regulate the expression of many genes, to identify key miRNA target genes and their functions can be a challenging task. Systems biology offers great promise for meeting these challenges by examining and inferring the regulatory, functional and positional importance of putative target genes and identifying the corresponding miRNAs regulating them. The systems perspective and network analysis can be of particular importance in studying diseases with complex molecular processes such as multiple cancer types.

In the present study, computational and systems biology approach have been adopted for the prediction of miRNAs from the ESTs of the *C. acuminata* and their targets in *Homo sapiens*; and based on the target gene list, further functional enrichment analysis, gene-disease associations and regulatory network analysis have been performed to identify important target genes, corresponding miRNAs regulating them, their role in disease development and underlying biological processes. Our study presents an extensive in silico analysis, which may pave a way forward to understand the cross-kingdom regulatory mechanism of plant-derived miRNAs with possibilities of regulating mammalian biological processes, molecular functions and associated pathways.

## 2. Results

### 2.1. Putative C. acuminata miRNAs

**Figure 1:**
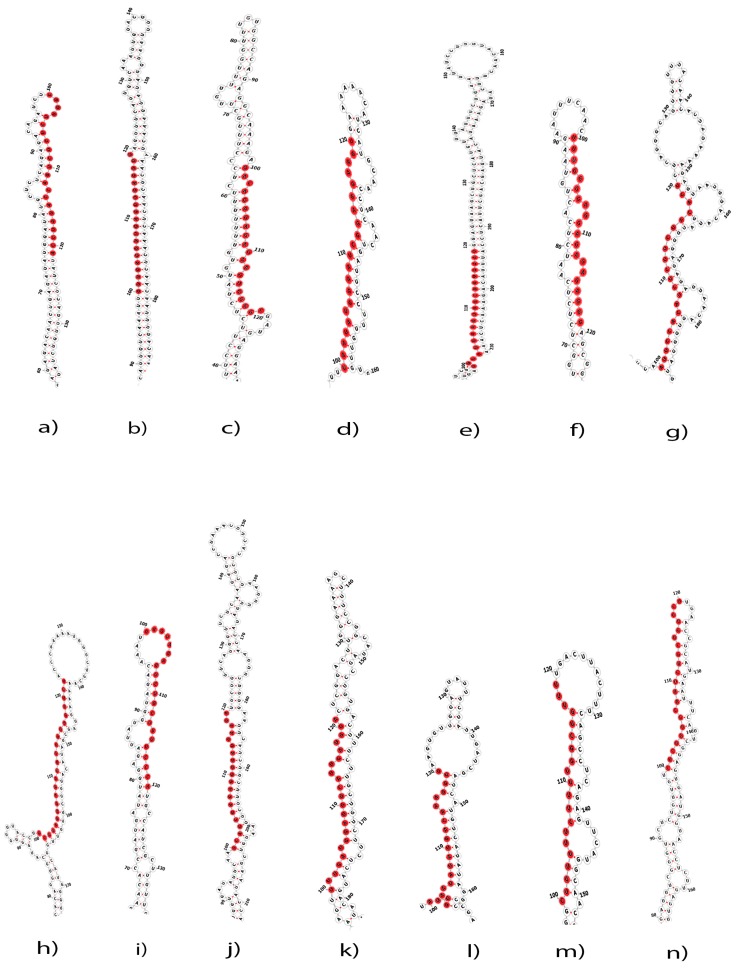
Predicted hairpin stem loop secondary structures of 14 putative miRNAs identified in *C. acuminate*. (**a**) cac-miR-3440-3p; (**b**) cac-miR-5653; (**c**) cac-miR-156e-3p; (**d**) cac-miR5049-3p; (**e**) cac-miR-5780d; (**f**) cac-miR-4723-3p; (**g**) cac-miR-7398f-5p; (**h**) cac-miR-548d-3p; (**i**) cac-miR-6903-5p; (**j**) cac-miR-7009-3p; (**k**) cac-miR-5291c; (**l**) cac-miR-5532; (**m**) cac-miR-29c-5p; (**n**) cac-miR-8157-3p; Mature sequences are marked with red color and the actual size of the precursors may be slightly longer than the one shown in the figure.

In the present study, initially to predict the putative miRNAs, total of 53,294 EST sequences of *C. acuminata* were screened based on the MFE (∆G) values, from which 213 miRNA precursor sequences were shortlisted. Further sorting was performed based on the criteria specified in methods section which resulted in 14 potential miRNAs (Table 1). Homologs of these computationally predicted miRNAs were observed in eleven different species, including two miRNAs each from *Brachypodium distachyon*, *Mus musculus* and *Salmo salar*. miRNA screened from EST medp_camac_20101112|6065 was found to be homologous with miRNA hsa-miR-4723-3p of *Homo sapiens* with an MFE of −56.3 kcal/mol. The predicted mature miRNAs varied in length ranging from 20 to 23 nucleotides. The minimum fold energy of the predicted miRNAs was found to be in the range of −55.09 kcal/mol to −102.70 kcal/mol corresponding to mmu-miR-7009-3p and ath-miR5653 respectively. The MFE is an important parameter for predicting the secondary structures of DNA and RNA. The lower value of MFE corresponds to the more thermodynamically stable secondary structures of DNA or RNA [44]. The MFEI value for each sequence was calculated as discussed in the method section. A candidate RNA sequence is considered to be a miRNA when its MFEI value is greater than 0.85 which is significantly higher than that for other types of RNAs like rRNAs (0.59), tRNAs (0.64) and mRNAs (0.62–0.66) [45]. The newly identified *C. acuminata* pri-miRNAs in our study were found having high MFEI ranging from −0.56 to −1.38 with an average of about −0.97, which can be considered as putative miRNAs. Mature miRNA sequences are found to be present in the stem portion of the hairpin structures (Figure 1). The predicted miRNA hairpin structure contains about 20−23 nucleotide engaged in G/U—pairings in the stem region without any large or internal loops or bulges.

### 2.2. Target Gene Identification and Their Functional Enrichment Analysis

As evident that most mature miRNAs regulate the expression of genes by binding to certain sites of target mRNAs in both plants and animals, a basic target prediction approach was considered to retain the maximum potential targets in human. Total 152 human genes were identified as targets of the predicted *C. acuminata* miRNAs (Table 2), which were used for the further analysis.

The functional enrichment analysis was applied to the predicted target genes. Out of 147 genes, three genes viz. *CRTAP*, *FAM49A* and *PRELP* remained functionally un-clustered. After analyzing the result, majority of the genes depicted roles in binding and cellular processes (Functional annotations of all 152 target genes have been provided in the supplementary file S3). Further classification of genes into protein classes showed their involvement in transcription factors, transporters and nucleic acid binding.

In functional annotation, three pathways with significant *p*-values (<0.05), were found to be associated with the target gene list. In our study, total 5 genes viz. *GSK3B*, *IGF1*, *ITGA2*, *PIK3R3* and *PARVA* were shown to be associated with “Focal Adhesion pathway” (*p*-value 0.021). While the “Regulation of lipolysis in adipocytes pathway” (*p*-value 0.034) included *ABHD5*, *NPY1R* and *PIK3R3* genes, the “mTOR signaling pathway” (*p*-value 0.036) showed the involvement of *RPS6KA6*, *IGF1* and *PIK3R3* genes. As evident, *PIK3R3* encoding Phosphatidylinositol 3-kinase regulatory subunit gamma enzyme was found to be involved in all three above mentioned pathways.

### 2.3. Gene Disease Associations

**Figure 2:**
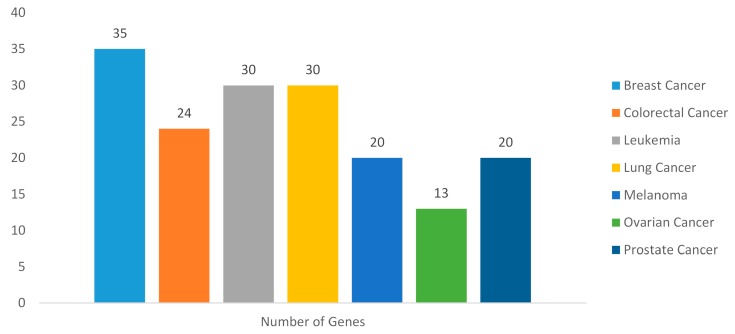
Gene-disease associations (with seven prominent cancer types).

Genes are important regulatory units and participate in almost all cellular activity. The mutational changes in genes can alter the functions of expressed proteins and can lead to the alteration of pathways, which can thereby result in the development and progression of different types of diseases. To find what kind of diseases are associated with these predicted target genes, gene-disease associations were mapped for all 152 target genes. A total 3468 gene-disease associations were obtained by mapping the target gene list to the database. Among the gene-disease associations, many diseases such as breast cancer, leukemia, lung cancer, retinoblastoma and arthritis were highly associated. After analysis and filtration of result based on the concept unique identifiers (CUIs), total 1537 disease terms were found to be associated with the target gene list (The supplementary file S1 shows detailed information for all of these gene-disease associations). By filtering the result further based on the term “cancer”, 70 genes were found to be associated with seven prominent types of cancers viz. Breast cancer, colorectal cancer, leukemia, lung cancer, melanoma, ovarian cancer and prostate cancer. Predominantly, maximum number of genes (35) are associated with breast cancer followed by 30 genes with leukemia and lung cancer while, 24 genes with colorectal cancer (Figure 2). In the present result, it was observed that a single gene is associated with different types of diseases as *ITGA2* is involved in arthritis, chronic kidney disease and eye disease.

### 2.4. Protein–Protein Interaction Network and Statistical Validation

After mapping target genes to their expressed proteins and extracting the interactions as described in the materials and methods section, the interaction data contain a total of 357 binary interactions between 367 proteins (nodes) and 39 protein complexes having 306 proteins involved in making complexes. To construct network, both types of data were combined and the final data was found to contain 624 proteins, with a total of 68,215 interactions. Further, network data was processed by Cytoscape and removed 58,320 multiple edges, 311 self-loops and 2 isolated nodes, resulting the protein network containing 622 nodes (proteins) and 9584 edges (interactions).

As mentioned in the method section, the KS-test was used to calculate the *p*-value to assess the significance of the network and its organization by comparing the degree distributions between the random and constructed networks using the Erdos-Renyi (ER) model of randomization. The *p*-value obtained thus were found to be very significant i.e., less than 2.2 × 10^−16^.

### 2.5. Hub Proteins and Centrality Parameters

**Figure 3:**
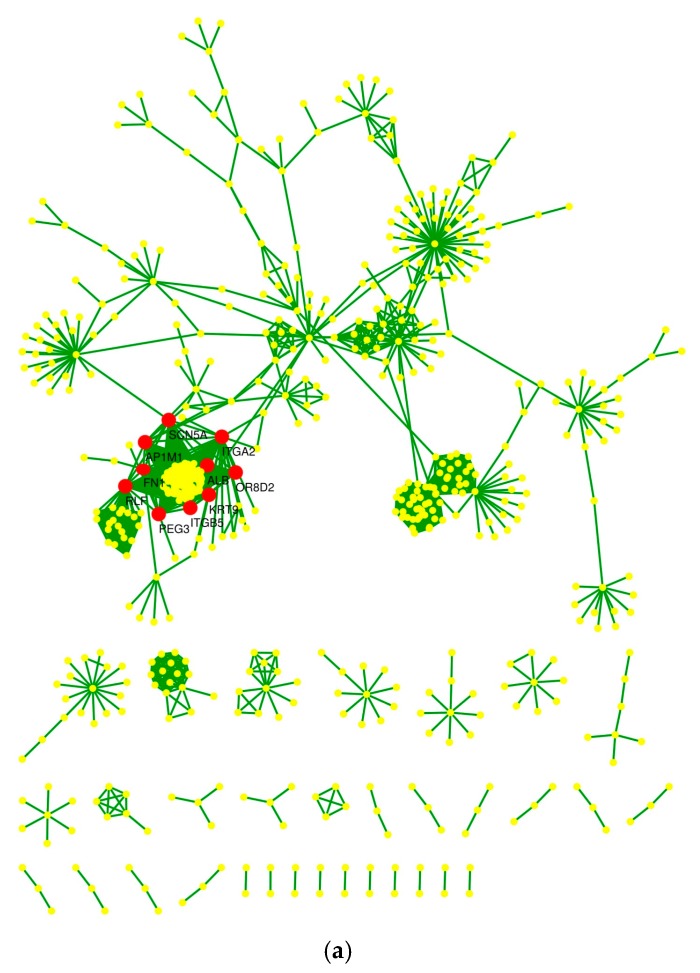

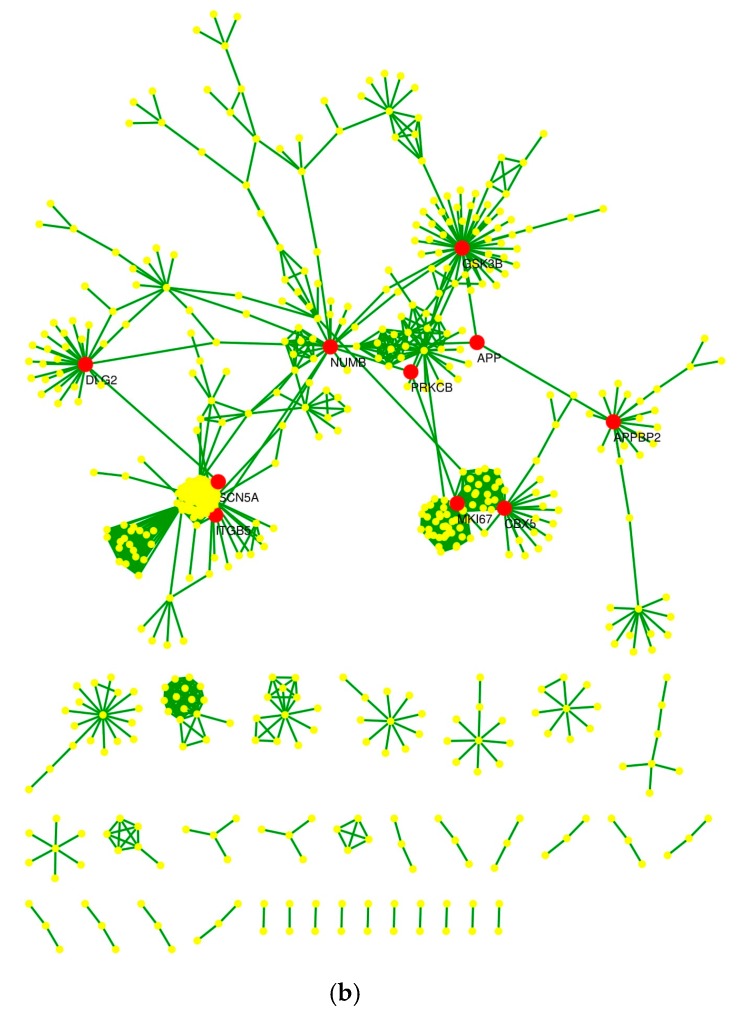
(**a**) Top 10 Hub nodes detected by Degree method, Top 10 hub nodes are marked with red color, which have been detected based on degree method; (**b**) Top 10 Hub nodes detected by Bottleneck method, Top 10 hub nodes are marked with red color, which have been detected based on bottleneck method.

Top ten hub nodes were detected and clustered using CytoHubba. ALB (Albumin), KRT9 (Keratin 9) and OR8D2 (Olfactory Receptor Family 8 Subfamily D Member 2) ranked first as evaluated by Degree method, whereas NUMB, Endocytic Adaptor Protein was detected as the top most hub node by Bottleneck method (Table 3 and Table 4). Two nodes ITGB5 (Integrin Subunit β 5) and SCN5A (Sodium Voltage-Gated Channel Alpha Subunit 5) were common in both the methods. Top 10 hub nodes detected by Degree and Bottleneck methods respectively have been labelled in the network (Figure 3a,b).

The Centrality parameters like Radiality, Betweenness, Degree, Stress, Eigen vector and Bridging were calculated to understand the nature and significance of nodes in the network and their underlying biological processes. Details of the calculated centrality parameters are available in Supplementary file S2. The top 10 nodes ranked by centrality values have been summarized in Table 5.

The calculated centrality parameters revealed many significant and biologically important proteins like NUMB, ITGB5, ITGA2, RLF, GSK3B and PRKCB from both experimental and topological point of view. Further, understanding the underlying biological processes and molecular functions of these proteins and corresponding genes will help to understand their regulatory mechanism, involved pathways and diseases. These hub and important nodes based on the centrality parameters may be considered as potential drug-targets for developing novel therapy for human disease like cancer and further can be validated experimentally. It was observed that the NUMB protein had high value of stress, betweenness and radiality. High stress and betweenness value suggested that it may be functionally capable of holding together communicating nodes and heavily involved in the cellular processes. High radiality depicted that the protein may act as organizing functional units or modules and can play a central role in regulation of the network. The node RLF found to have highest eigen vector and degree value in the network indicating that it could be a critical target of the regulatory pathway and may be playing a central role in the regulation of other important proteins. The node PRKCB found to have high bridging value, which means that it may be a regulatory protein interacting with several other important proteins via clusters or dense region of the network.

## 3. Discussion

MiRNAs are emerging as potential gene managers that play a key role in regulation of molecular pathways. A thought-provoking hypothesis on plant-derived miRNAs by Zhang et al. [16] came in 2012. According to their findings, food derived miRNAs could enter into body fluid and tissues of animals and can regulate expression of endogenous mRNAs. Plenty of studies have also shown that plant-derived miRNAs may transfer and regulate functional expression of genes across different species [12,46]. These studies on medicinal plants and miRNAs in various cancers have given rise of new cancer therapies, for example, the up-regulation of miR-23a expression may regulate *p53* involved in hepatocellular carcinoma (HCC) [47]. Thus, plant miRNAs may act as regulatory factors in the human body system by post-transcriptional regulation of endogenous mRNAs as medicinal plants will be used to treat cancers. The medicinal plant derived miRNAs may give rise to a vowing hypothesis of the cross-kingdom mechanism of action of medicinal plants for cancer therapy, but more experimental evidences need to support the therapeutic application of herbal miRNAs [35]. Assuming the vast possibility of miRNA as an alternate therapeutic option, one of the main objectives of miRNA research is to identify the miRNA-mRNA interactions and functional enrichment analysis of target genes to investigate their regulatory and functional roles. The miRNA-based therapeutics may prove to be promising at targeting tumor cells directly or as complementary of other therapies, for example, to overcome the drug-resistance issue of tumor cells.

Since *C. acuminata* is a medicinal plant having anti-cancer activity, miRNAs derived from it may potentially regulate cancer related pathways. A study revealed that Camptotheca extracted from the stem and bark of *C. acuminata* down-regulated the expression of miR-125b and activated the apoptosis pathways in mitochondria by up-regulating the expression of the *p53*, *Mcl1*, and *Bak1* genes and induced apoptosis in cancer cells [48]. Computational approaches using Expressed sequence tags (EST) have been extensively used in previous studies for the prediction and discovery of new miRNAs and their targets and this method of identifying putative miRNAs is advantageous in case of unavailability of whole genome sequence [49,50,51,52]. In our study, 14 miRNAs were predicted from 53,294 ESTs possibly regulating 152 genes in the human system. Out of these 14 miRNAs, cac-miR-4723-3p is homologs of human and miR-4723 has been proved to inhibit growth, proliferation and metastasis in prostate cancer cell lines. The overexpressing miR-4723 in these cell lines was reported to be negatively regulating Integrin, alpha 3 (*ITGA3*), Abelson proto-oncogene and *MeCP2* (Methyl CpG binding protein) genes [53].

In our study, the target genes were found to be associated with focal adhesion, regulation of lipolysis in adipocytes and mTOR signaling pathways through KEGG pathways analysis. Our result showed the involvement of *ITGA* and *ITGB* in the Focal adhesion pathway. The Focal adhesion kinase (FAK), an important component of the focal adhesion pathway is a non-receptor tyrosine kinase found in cytoplasm that play important role in integrin mediated signaling and a study on the focal adhesion kinase and the pathway has indicated its role in cancer progression and angiogenesis [54]. Further, the alterations in major components of the mTOR pathway like loss of function of *PTEN*, *PI3K* amplification/mutation and over-expression of *S6K1*, *4EBP1*, *eIF4E* and *AKT* have been reported to be associated with many types of cancers, particularly in melanoma and glioblastama [55,56]. While, the decreased levels of lipogenic enzymes Lipoprotein lipase (LPL) and Fatty acid synthase (FAS), important components of the regulation of lipolysis in adipocytes pathway was reported to be associated with colorectal cancer [57].

Functional clustering of these genes showed that many of them encoded plasma membrane proteins. Presence of circulating miRNAs is well acknowledged in human plasma, serum and blood cells and may function as mediators of cell communication [26,58], besides being endocytosed into intestinal basolateral membrane cells [59]. Not surprisingly, the predicted miRNAs seem to regulate genes encoding proteins, which are cellular components of plasma membrane, basolateral plasma membrane and recycling endosomal membrane, as depicted in results. When the molecular functional annotation was done, 81 genes were discovered to be involved in protein binding.

Further, in the protein network analysis, several important proteins like ITGA2, NUMB, PEG3, GSK3B, ATP2B4, MKI67, DLG2, BTRC, CBX5 and APPBP2 among all proteins encoded by all 152 target genes were identified based on hub nodes detection and centrality parameters like radiality, betweenness, degree, stress, eigen vector and bridging, whose functional role have been reported in disease related pathways. The proteins encoded by *ITGA2* (integrin alpha-2) and *ITGB5* (integrin beta-5) genes were found to be among the top 10 hub proteins. It has been well studied that Hub proteins such as, oncogenes *TP53* and *SRC* are well known hallmarks of tumorigenesis [60]. A recent study on *ITGA2* showed that the miR-373 represses the expression of *ITGA2* and stimulates the migration of breast tumor cells [61]. While, another study on *ITGB5* showed its crucial role in the tumorigenesis of breast tumor cells [62]. Thus, *ITGA2* and *ITGB5* can be considered as therapeutic biomarkers for the prognosis of breast cancer. Based on target prediction result, it has been found that *ITGA2* is the target of miR-29c-5p. A recent report by Yi-Jun Shu et al. (2017) suggested that miR-29c-5p has a tumor-suppressive nature that may act as potential biomarker for prognostic purpose of gallbladder cancer [63].

It was observed that the down-regulation of *NUMB* suppressed cell growth and found to be associated with cancers like liver [64], leukemia [65] and lung cancer [66]. Besides that, it negatively regulates the Notch signaling pathway [67]. An in vivo experiment provides the first evidence for the functional involvement of *NUMB* in the inactivation of Notch by stimulating its endocytosis [68]. While, previous studies had shown the involvement of the Notch signaling pathway in the tumorigenesis of several malignancies like non-small cell lung cancer [69], medulloblastoma [70], Kaposi’s sarcoma [71], ovarian cancer [72] and melanoma [73]. It has been evidenced that acquired chemo-resistance in ovarian cancer is associated with the epigenetic silencing of microRNA-199b-5p via activation of JAG1-Notch1 signaling pathway [74]. A study on *Mus musculus* exhibited that the activation of Notch signaling pathway by breast cancer cells expressing Jagged1, stimulates the metastasis of bone in bone microenvironment [75]. The target prediction result revealed that *NUMB* can be targeted by miR-5780d. The *PEG3* (Paternally expressed gene 3) is a DNA-binding protein encoding gene and has vital role in fetal growth rate control [76]. The contribution of *PEG3* has been evident in pathogenesis of ovarian cancer through loss of its expression and promoter methylation [77]. Assuming the role of *PEG3* in *p53*-mediated apoptosis, there is possibility that *PEG3* may function as a tumor suppressor in case of ovarian cancer [78]. The target prediction result showed that the expression of *PEG3* can be regulated by miR-29c-5p. The *GSK3B* (Glycogen synthase kinase-3 beta) is a negative regulator of glycogenesis found regulating various cellular functions and signaling pathways [79]. It has been investigated that the inhibition of *GSK3B* induces cell death including mitotic devastation by dysregulation of centrosome in tumor cells [80]. Based on our analysis result, it was found that miR-548d-3p can regulate the expression of *GSK3B*. The *ATP2B4* gene encodes one of the four isoforms of p-type ATPase PMCA enzyme and bears critical importance in maintaining the balance of intracellular calcium homeostasis by providing the export of calcium ions out of the cell. The *MKI67* (Marker of Proliferation Ki-67) gene is a nuclear protein coding gene associated with cellular proliferation. An experiment on *MKI67* indicated that the cellular growth of hepatocellular carcinoma can be suppressed by targeting *MKI67* through miR-519d [81]. *DLG2* (Discs Large MAGUK Scaffold Protein 2), a protein coding gene, is a member of the MAGUK (membrane-associated guanylate kinase) family. The *DLG2* is associated with renal cancer [82]. *BTRC* (Beta-Transducin Repeat Containing E3 Ubiquitin Protein Ligase) is a protein coding gene. The protein encoded by *BTRC* is a member of the F-box protein family and disease associated with *BTRC* is Shigellosis [83]. Based on our analysis result, it was found that the expression of *MKI67*, *DLG2* and *BTRC* might be regulated by miR-4723-3p. The *CBX5* (chromobox homolog 5) is a non-histone protein coding gene. A recent experiment on *CBX5* identified it as a putative target in lung cancer through a scalable network-based target identification process [84]. Based on our analysis result, it was found that miR-7009-3p can regulate the expression of *CBX5*. The *APPBP2* (Amyloid Beta Precursor Protein Binding Protein 2) is a protein coding gene and its overexpression is associated with Ovarian clear cell adenocarcinoma [85]. Based on our analysis result, we can say that the expression of *APPBP2* can be regulated by miR-5653.

## 4. Materials and Methods

### 4.1. Data Retrieval

**Figure 4:**
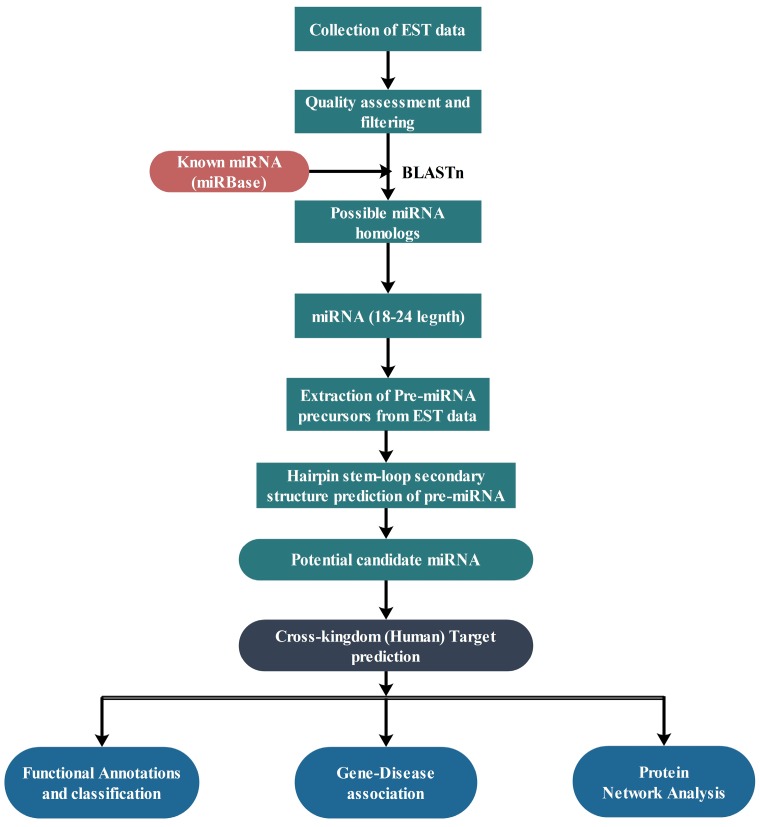
Brief workflow of the study.

Total 53,294 ESTs of *C. acuminata* were extracted from the medicinal plant database (MedPlant|RNA Seq Database) [86]. For sequence homology studies, all of these ESTs were screened against the known miRNAs available in miRBase v21 [87]. To identify potential miRNAs, miRBase Release 21 containing 28,645 entries corresponding to hairpin precursor miRNAs and 35,828 expressed mature miRNAs in total 223 species was considered for the analysis. These miRNAs were taken as a reference set of miRNA sequences to predict the putative novel miRNAs from the ESTs of *C. acuminata*. Figure 4 shows a brief workflow of the study.

### 4.2. Prediction of Putative Novel miRNAs

Blastn was performed against the extracted miRBase miRNA datasets to screen out the candidate miRNAs taking E-value 1E-5, word length of 6 and percent identity of 80 with a maximum mismatch of 3. A total of 53,294 ESTs of the *C. acuminata* were screened in this process. Further, miRNA homologs having 18–24 nucleotide in length were screened out. In next step, all repeat sequences were removed and 4722 miRNA homologs were obtained. To remove protein coding sequences, Blastx was performed against plant protein database and a total of 2359 non-coding sequences were obtained after removing protein coding sequences. Based on the secondary structure prediction criteria and minimum fold energy index (MFEI), putative miRNAs were predicted.

### 4.3. Prediction of Secondary Structure

The unique reference miRNA sequences were mapped on ESTs of *C. acuminata*. Flanking region of 100 nucleotide base pair upstream and 100 nucleotide base pair downstream were extracted using an in-house script. RNAFold version 1.8.4 from Vienna RNA package [88] was used to predict secondary structures to find out the minimum free energy structures. Structural analysis was done on the basis of the following criteria for the confirmation of miRNAs: (a) Formation of stem-loop hairpin secondary structure; (b) presence of less than 3 nucleotide substitutions in predicted mature miRNAs as compared with the all known miRNAs extracted from miRBase; (c) miRNA sequences without any loop and break and (d) Minimum Fold Energy Index (MFEI); (e) Presence of mature sequence on single arm of the stem loop. The MFEI was calculated according to the following equation reported by Zhang et al.:

MFEI = [(Minimum Fold Energy/Length of the precursor RNA sequence) × 100]/(G + C) %
(1)

whereas, Minimum Fold Energy represented by (ΔG) values.

miRNA precursor sequences used to have significantly higher negative MFEs and MFEIs in comparison to other types of RNAs e.g., tRNAs, rRNAs [89,90].

### 4.4. Nomenclature of miRNAs

The nomenclature of newly predicted miRNAs were implemented as per the miRBase database. The mature miRNAs were labeled as “miR” with the prefix “cac” for *C. acuminata*.

### 4.5. Prediction of Potential Targets of miRNAs

The target genes for the newly predicted miRNAs from the *C. acuminata* in *Homo sapiens* were identified by a perfect or near-perfect complementarity between miRNA and its target transcript and the target site accessibility by calculating unpaired energy (UPE) necessary for opening the secondary structure around the miRNA target site. The mature miRNA sequences were used as the query sequences for psRNATarget [91] prediction webservers against *Homo sapiens* with parameters as: (1) Maximum expectation value 3; (2) multiplicity of target sites 2; (3) range of central mis-match for translational inhibition 9–11 nucleotides; (4) maximum mismatches at the complimentary site ≤ 4 without any gaps.

### 4.6. Functional Analysis of Target Genes

Functional enrichment analysis for the predicted target genes was performed using DAVID (Database for Annotation, Visualization and Integrated Discovery) [92] with default parameters. The GO terms (Biological process, Cellular components and Molecular functions) were considered to be statistically significant with threshold *p*-value < 0.05. Kyoto Encyclopedia of Genes and Genomes (KEGG) [93] pathways mapping with *p*-values < 0.05 of DAVID was investigated to infer the role of target genes of predicted miRNAs in different pathways and their associations in various diseases.

### 4.7. Gene-Disease Associations

The predicted miRNA target genes may be involved in the development and progression of several diseases. To find what kind of diseases are associated with these predicted target genes, gene-disease association analysis was performed on the basis of target gene list. All the target genes were mapped against the DisGeNET database [94], in order to understand the associations wherein one gene may be involved in several diseases and vice versa may also hold true. The DisGeNET is a database on human gene-disease associations integrated from various curated databases and the literature based text-mining information.

### 4.8. Protein-Protein Interaction Data and Network Construction

As one miRNA can regulate a large number of genes, it can be a challenging task to identify key miRNA target genes and their functions from the long list of target genes. The regulatory network analysis can be used to infer the functional importance of putative target genes and the corresponding miRNAs regulating them.

In order to construct the network of proteins of corresponding miRNA target genes, all these target genes were mapped to the UniProt database [95], which resulted in a list of all proteins expressed by them. Further, the protein interaction data was extracted from the Human Protein Reference Database (HPRD), Release 9 [96]. This database contains two types of interaction data-Binary, if two proteins interact directly and Complex, when several proteins form a complex. As protein complexes are functional group and participate in activation or inhibition, these complexes were also included in our study with the assumption that each protein is in a complex interaction with one another. Thus, the extracted interaction data contained both types of data: binary interactions and protein complexes. The protein-protein interaction network was constructed and visualized using Cytoscape software [97].

### 4.9. Statistical Validation Using Network Randomization

The significance of the resultant protein network was evaluated by comparing its degree distributions with the degree distributions of the corresponding randomized networks. For this purpose, the Erdos-Renyi model (ER model) of randomization was implemented to simulate networks 10,000 times. The randomization study by this model was performed using igraph R package [98]. Kolmogorov–Smirnov (KS) test was used to compare the actual network with negative control to calculate the *p*-value. The significance level was set at *p* < 0.05.

### 4.10. Regulatory Network Analysis

Hub nodes and clusters present in the protein network were detected using CytoHubba [99], a Cytoscape app. Furthermore, the protein network was evaluated on various centrality parameters using CentiScaPe [100] to assess its property, significance and organization to understand their underlying biological processes. The centrality parameters which have been studied in the present study are (Some notations before describing parameters: The shortest path length between nodes *u* and *v* is denoted as dist(*u*,*v*). Let *C*(*v*) be the component of the network, which contains node *v*. The dist(*u*,*v*) is equal to infinite if *C*(*v*) ≠ *C*(*w*)):

Radiality (Rad): The radiality is a node centrality index. The radiality of a node *v* is the summation of the subtracted values of all shortest paths between the node *v* and all other nodes in the network and the value of the diameter + 1 and then divided by the number of nodes − 1 [101].

Rad(*v*) = |*V*(*C*(v))||*V*| × ∑*w* ∈ *C*(*v*) (Δ*C*(*v*) + 1 − dist(*v*,*w*))max{dist(*v*,*w*):*w* ∈ *C*(*v*)} (2)

where Δ*C*(v) is the maximum distance between any two vertices of the component *C*(*v*).

Betweenness (BC): The Betweenness is a node centrality index. The betweenness of a node *v* is the total number of shortest paths between nodes *s* and *t* passing through a node *v* i.e. σ*st*(*v*) divided by the total number of shortest paths between nodes *s* and *t* i.e., σ*st* [102].

BC(*v*) = ∑*s* ≠ *t* ≠ *v* ∈ *C*(*v*)σ*st*(*v*)/σ*st* (3)

where, σ*st* is the number of shortest paths from node *s* to node *t*.

Degree (Deg): The degree is the simplest centrality index of a node. It is the total number of nodes directly connected to a given node *v* [103].

Deg(*v*) = |*N*(*v*)| (4)

Stress (Str): The stress is a node centrality index. The stress of a node *v* is the all shortest paths in a network passing through node *v* [104].

Str(*v*) = ∑*s* ≠ *t* ≠ *v* ∈ *C*(*v*)σ*st*(*v*) (5)

where, σ*st*(*v*) is the number of shortest paths from node s to node *t* which use the node *v*.

Eigen vector (Ev): The eigen vector centrality is a node centrality index. It is a weighted sum of all direct as well as indirect connections of different length by taking into account the whole network The eigen vector centrality of a node is proportional to the sum of the centralities of all its connected node [105].

Let *G*(*E*,*V*) be a graph, with *V* vertices and *E* edges. Thus, *A* = adjacency matrix; If two vertices are connected, $a_{ij}$ = 1 and if not $a_{ij}$ = 0.

(6) $$Ax=\lambda x,\;\lambda x_{i}=\sum_{j=1}^{n}a_{ij}x_{j},\;i=1,\ldots ,n$$

where, *λ* = largest eigenvalue of *A* and *n* = number of vertices.

Bridging (Brg): The bridging is a node centrality index. The bridging of a node is calculated by multiplying its bridging coefficient (BCoeff) and the betweenness centrality (BC). If a node has high degree neighbors, its bridging coefficient is high [106].

Brg = BCoeff × BC
(7)

## 5. Conclusions

The present study shows the importance of predicted miRNAs of *C. acuminata* targeting human genes through functional annotation and network analysis of these target genes. The annotation of these genes showed their association with pathways which may be involved in cancer progression. Thus, these putative microRNAs hold significant potential value in terms of regulating gene expression and disease progression including cancer. Plant based miRNAs may lead to the development of new miRNA based therapeutics by further elucidating the regulatory mechanism of miRNA by applying unbiased computational and systems biology approach followed by evidence based molecular validations. Thus, our study supports the promising hypothesis of cross-kingdom regulation by plant derived miRNAs. It also demonstrates the possible role of miRNAs in regulating complex disease networks in the human system. Although these genes may regulate other pathways, in the present study a strong association is observed with cancer pathways. Based on the in silico systems biology studies, further extensive molecular validations are required to establish a clear relationship between these miRNAs and cancer associated pathways. Even though it remains to be a challenge, the approach however will facilitate and open up immense opportunities towards the development of potential novel miRNA based therapeutics.

## Figures and Tables

**Table 1:** List of 14 putative miRNAs identified in *C. acuminate.*

Sr. No	EST ID	miRNA Name	Homolog miRNA	Mature Sequence	MSL	PSL	MFE (ΔG)	MFE in Kcal/mol	(G + C) %	MFEI
1	medp_camac_20101112|9453	cac-miR-5653	ath-miR5653	GTTGAGTTTGAGTTGAGTTG	20	205	−100	−102.7	35.12	−1.389
2	medp_camac_20101112|7558	cac-miR-5780d	gma-miR5780d	TGTTTTGAGTTTCTG-TAAAT	21	210	−80.8	−83.96	32.86	−1.171
3	medp_camac_20101112|10526	cac-miR-3440-3p	aly-miR3440-3p	CGGTTCTCTCTGACCATATCCA	22	141	−74.1	−75.88	45.39	−1.158
4	medp_camac_20101112|33501	cac-miR-8157-3p	ssa-miR-8157-3p	CTCTGTGCATTCTGCTGTGCT	21	220	−67.7	−72.58	52.27	−0.589
5	medp_camac_20101112|6119	cac-miR-6903-5p	mmu-miR-6903-5p	TGGTAGAGT-CTGCTTTTCCCA	22	220	−61.3	−64.88	40.45	−0.689
6	medp_camac_20101112|44664	cac-miR-7398f-5p	mdo-miR-7398f-5p	ATT-CCACATCTCTTCTACACT	22	220	−60.4	−64.34	44.09	−0.623
7	medp_camac_20101112|9293	cac-miR-156e-3p	bdi-miR156e-3p	GACAGAGAGAGAAGTGGAGC	20	183	−58.5	−61.48	42.08	−0.760
8	medp_camac_20101112|29	cac-miR-5532	osa-miR5532	ATGGAATATATGACAAAGGTG	21	220	−57.4	−61.99	39.09	−0.667
9	medp_camac_20101112|6065	cac-miR-4723-3p	hsa-miR-4723-3p	TTTGGGGAGGAG--AGAGAGGG	22	217	−56.3	−61.33	45.16	−0.574
10	medp_camac_20101112|447	cac-miR-5049-3p	bdi-miR5049-3p	TAATATGGAATCGGAGGAAGT	21	220	−53.3	−57.51	39.55	−0.613
11	medp_camac_20101112|3541	cac-miR-5291c	mtr-miR5291c	TTTGATGGATGGCATTG-ATGGA	23	221	−53.2	−57.84	41.63	−0.578
12	medp_camac_20101112|4893	cac-miR-548d-3p	mml-miR-548d-3p	GCAGAAAGAAATTGTGGTGTTTT	23	222	−53.1	−58.01	37.39	−0.640
13	medp_camac_20101112|18253	cac-miR-29c-5p	ssa-miR-29c-5p	CTGTTTTCTTTTGGCTGTTT	20	219	−52.2	−56.62	42.47	−0.561
14	medp_camac_20101112|10789	cac-miR-7009-3p	mmu-miR-7009-3p	GCAGGGAGAGGGGATAAAGA	20	219	−51.4	−55.09	36.99	−0.635

EST: Expressed Sequence Tag; MSL: Mature Sequence Length; PSL: Precursor Sequence Length; MFE: Minimum Fold Energy; MFEI: Minimum Fold Energy Index.

**Table 2:** Potential target genes of 14 miRNAs identified in *Homo sapiens.*

Sr. No	miRNA Name	miRNA_Acc.	Target Gene
1	cac-miR-3440-3p	medp_camac_20101112|10526	*CCNJL*
2	cac-miR-7009-3p	medp_camac_20101112|10789	*GPATCH8*, *POM121C*, *TMEM14E*, *ZDHHC3*, *IYD*, *IYD*, *FCRLA*, *CLCN6*, *PIP4K2B*, *ARPP19*, *CBX5*, *MGAT4A*, *HS2ST1*, *CEP350*, *ZNF609*, *DSTYK*, *BNC2*, *KANSL2*, *BCL11A*, *HAUS3*, *HMBOX1*, *SOX7*, *DIDO1*, *MUM1L1*, *APCDD1*, *POM121*, *ANKRD52*, *COX18*, *TDRD1*, *IYD*, *PPAPDC2*
3	cac-miR-29c-5p	medp_camac_20101112|18253	*ZNF37A*, *SOGA3*, *ARGFX*, *CELF2*, *CELF2*, *SPATA6L*, *PIK3R3*, *METTL20*, *RPS6KC1*, *TTC26*, *POLR3B*, *FBXL17*, *MEF2C*, *CD44*, *CCR1*, *ITGA2*, *KCNJ10*, *AKAP5*, *PEG3*, *CDC42EP3*, *SCN8A*, *RNF144A*, *ABHD5*, *TFB1M*, *RHOU*, *TMEM106C*, *GINS4*, *MRPL11*, *MRPL11*, *GPATCH4*, *ZNF169*
4	cac-miR-5532	medp_camac_20101112|29	*EXOSC3*, *KIF13A*, *EXOSC3*, *C11orf87*
5	cac-miR-8157-3p	medp_camac_20101112|33501	*C3orf18*, *RBM33*
6	cac-miR-5291c	medp_camac_20101112|3541	*GLIS3*, *CNOT11*, *C11orf87*
7	cac-miR-7398f-5p	medp_camac_20101112|44664	*NCOA4*, *NCOA4*, *STAMBP*
8	cac-miR-5049-3p	medp_camac_20101112|447	*CLHC1*, *CACUL1*, *MMAA*
9	cac-miR-548d-3p	medp_camac_20101112|4893	*ATP2B4*, *ATP2B4*, *GSK3B*, *MTUS2*
10	cac-miR-4723-3p	medp_camac_20101112|6065	*IGF1*, *GRINA*, *GPNMB*, *CREBZF*, *FAM3C*, *C1orf226*, *PHF21A*, *IGF1*, *SERPINA1*, *DLG2*, *SYTL4*, *DCTN5*, *TGOLN2*, *TGOLN2*, *UNC5B*, *B4GALNT1* *CPM*, *MKI67*, *PRELP*, *RGS7*, *SLC1A2*, *CBFA2T3*, *CRTAP*, *TGOLN2*, *MYO9A*, *EID1*, *RPS6KA6*, *ABLIM3*, *ZBTB7A*, *PACS1*, *PARVA*, *PCDHA1*, *PLEKHH1*, *PRX*, *RAP2C*, *EBF1*, *RNF39*, *C6orf25*, *MARVELD1*, *ANKRD27*, *DCTN5*, *EAF1*, *ITPRIP*, *BTRC*, *STK35*, *C6orf25*, *C6orf25*, *DBF4B*, *RNF39*, *SLC41A1*, *GALNT10*, *FMNL3*, *BACH1*, *PATE2*
11	cac-miR-6903-5p	medp_camac_20101112|6119	*SLC30A4*, *TRIM62*
12	cac-miR-5780d	medp_camac_20101112|7558	*NPY1R*, *NUMB*, *GLIS3*, *DCTN4*, *C11orf93*, *ZNF540*, *TIPARP*, *STARD13*, *STARD13*, *PROX1*, *MIP*, *UBXN4*, *SERTAD2*, *CLIP3*, *HPCAL4*, *CCDC93*, *FAM49A*
13	cac-miR-156e-3p	medp_camac_20101112|9293	*DDX17*, *KAZN*, *ASB4*, *PGLYRP4*, *JPH3*, *EPG5*, *TPCN2*
14	cac-miR-5653	medp_camac_20101112|9453	*LOC100996485*, *GYPA*, *APPBP2*, *EFR3B*, *RAP2A*, *TRUB1*, *NAP1L5*

152 genes identified in Human as potential targets of *C. acuminata* miRNAs.

**Table 3:** Top 10 Hub nodes detected by Degree method.

Rank	Protein	Score
1	ALB	2056
1	KRT9	2056
1	OR8D2	2056
4	ITGA2	1047
4	RLF	1047
6	AP1M1	1032
7	PEG3	1030
8	ITGB5	1029
8	SCN5A	1029
8	FN1	1029

**Table 4:** Top 10 Hub nodes detected by Bottleneck method.

Rank	Protein	Score
1	NUMB	428
2	ITGB5	394
3	GSK3B	122
4	APP	93
5	PRKCB	76
6	MKI67	75
7	SCN5A	74
8	DLG2	73
9	CBX5	31
9	APPBP2	31

**Table 5:** The top 10 proteins ranked by centrality values.

Rank	Radiality	Betweenness	Degree	Stress	Eigen Vector	Bridging
1	NUMB	NUMB	RLF	NUMB	RLF	PRKCB
2	ITGB5	ITGB5	ITGA2	GSK3B	ITGA2	APP
3	PRKCB	GSK3B	AP1M1	ITGB5	AP1M1	DPYSL2
4	SCN5A	PRKCB	PEG3	PRKCB	PEG3	NOTCH1
5	APP	MKI67	ATM	MKI67	ITGB5	CREB3
6	ITGB3	DLG2	FN1	APP	FN1	MDM2
7	RLF	APP	ITGB5	DLG2	SCN5A	PRKCA
8	AP1M1	SCN5A	SCN5A	BTRC	ATM	PRKAR2A
9	ITGA2	APPBP2	ADRA1B	SCN5A	ADRA1B	DLG4
10	FN1	BTRC	AGA	CBX5	AGA	ATP2B4

This list of proteins includes core proteins (expressed by target genes) as well as their interacting partners.

## Supplementary Materials

Supplementary materials can be found at www.mdpi.com/1422-0067/18/6/1191/s1.
