# Supplementary material for: Cross-Kingdom Regulation of Putative miRNAs Derived from Happy Tree in Cancer Pathway: A Systems Biology Approach
Source: Int J Mol Sci. 2017 Jun 3;18(6):1191. doi: 10.3390/ijms18061191 (PMC5486014; doi:10.3390/ijms18061191)
Supplement: Supplementary file 1 [file ijms-18-01191-s001.zip › Supplementary files/Supplementary file S3-Functional annotation.docx]

| **Sr. No** | **Gene** | **Functional annotation** |
| --- | --- | --- |
| 1 | ABHD5 | abhydrolase domain containing 5; Lysophosphatidic acid acyltransferase which functions in phosphatidic acid biosynthesis. May regulate the cellular storage of triacylglycerol through activation of the phospholipase PNPLA2. Involved in keratinocyte differentiation |
| 2 | ABLIM3 | actin binding LIM protein family, member 3; May act as scaffold protein. May stimulate ABRA activity and ABRA-dependent SRF transcriptional activity |
| 3 | AKAP5 | A kinase (PRKA) anchor protein 5; May anchor the PKA protein to cytoskeletal and/or organelle-associated proteins, targeting the signal carried by cAMP to specific intracellular effectors. Association with to the beta2-adrenergic receptor (beta2-AR) not only regulates beta2-AR signaling pathway, but also the activation by PKA by switching off the beta2-AR signaling cascade |
| 4 | ANKRD27 | ankyrin repeat domain 27 (VPS9 domain); May be a Rab21 guanine exchange factor and regulate endosome dynamics (By similarity) |
| 5 | ANKRD52 | ankyrin repeat domain 52; Putative regulatory subunit of protein phosphatase 6 (PP6) that may be involved in the recognition of phosphoprotein substrates |
| 6 | APCDD1 | adenomatosis polyposis coli down-regulated 1; Negative regulator of the Wnt signaling pathway. Inhibits Wnt signaling in a cell-autonomous manner and functions upstream of beta-catenin. May act via its interaction with Wnt and LRP proteins. May play a role in colorectal tumorigenesis |
| 7 | APPBP2 | amyloid beta precursor protein (cytoplasmic tail) binding protein 2; May play a role in intracellular protein transport. May be involved in the translocation of APP along microtubules toward the cell surface |
| 8 | ARGFX | arginine-fifty homeobox; Putative transcription factor (By similarity) |
| 9 | ARPP19 | cAMP-regulated phosphoprotein, 19kDa; Protein phosphatase inhibitor that specifically inhibits protein phosphatase 2A (PP2A) during mitosis. When phosphorylated at Ser-62 during mitosis, specifically interacts with PPP2R2D (PR55-delta) and inhibits its activity, leading to inactivation of PP2A, an essential condition to keep cyclin-B1-CDK1 activity high during M phase. May indirectly enhance GAP-43 expression |
| 10 | ASB4 | ankyrin repeat and SOCS box containing 4; Probable substrate-recognition component of a SCF-like ECS (Elongin-Cullin-SOCS-box protein) E3 ubiquitin-protein ligase complex which mediates the ubiquitination and subsequent proteasomal degradation of target proteins. Promotes differentiation and maturation of the vascular lineage by an oxygen-dependent mechanism (By similarity) |
| 11 | ATP2B4 | ATPase, Ca++ transporting, plasma membrane 4; This magnesium-dependent enzyme catalyzes the hydrolysis of ATP coupled with the transport of calcium out of the cell |
| 12 | B4GALNT1 | beta-1,4-N-acetyl-galactosaminyl transferase 1; Involved in the biosynthesis of gangliosides GM2, GD2 and GA2 |
| 13 | BACH1 | BTB and CNC homology 1, basic leucine zipper transcription factor 1; Transcriptional regulator that acts as repressor or activator. Binds, in vitro, to NF-E2 binding sites. Play important roles in coordinating transcription activation and repression by MAFK |
| 14 | BCL11A | B-cell CLL/lymphoma 11A (zinc finger protein) |
| 15 | BNC2 | basonuclin 2; Probable transcription factor specific for skin keratinocytes. May play a role in the differentiation of spermatozoa and oocytes |
| 16 | BTRC | beta-transducin repeat containing E3 ubiquitin protein ligase; Substrate recognition component of a SCF (SKP1-CUL1-F- box protein) E3 ubiquitin-protein ligase complex which mediates the ubiquitination and subsequent proteasomal degradation of target proteins. Recognizes and binds to phosphorylated target proteins. SCF(BTRC) mediates the ubiquitination of CTNNB1 and participates in Wnt signaling. SCF(BTRC) mediates the ubiquitination of NFKBIA, NFKBIB and NFKBIE; the degradation frees the associated NFKB1 to translocate into the nucleus and to activate transcription. Ubiquitination of N [...] |
| 17 | C11orf87 | chromosome 11 open reading frame 87 |
| 18 | C11orf93 | chromosome 11 open reading frame 93 |
| 19 | C1orf226 | chromosome 1 open reading frame 226 |
| 20 | C2orf29 | chromosome 2 open reading frame 29; Component of the CCR4-NOT complex which is one of the major cellular mRNA deadenylases and is linked to various cellular processes including bulk mRNA degradation, miRNA-mediated repression, translational repression during translational initiation and general transcription regulation. Additional complex functions may be a consequence of its influence on mRNA expression. Is required for the association of CNOT10 with the CCR4-NOT complex. Seems not to be required for complex deadenylase function |
| 21 | C3orf18 | chromosome 3 open reading frame 18 |
| 22 | C6orf25 | chromosome 6 open reading frame 25 |
| 23 | CACUL1 | CDK2-associated, cullin domain 1; Cell cycle associated protein capable of promoting cell proliferation through the activation of CDK2 at the G1/S phase transition |
| 24 | CBFA2T3 | core-binding factor, runt domain, alpha subunit 2; translocated to, 3 |
| 25 | CBX5 | chromobox homolog 5; Component of heterochromatin that recognizes and binds histone H3 tails methylated at 'Lys-9' (H3K9me), leading to epigenetic repression. In contrast, it is excluded from chromatin when 'Tyr-41' of histone H3 is phosphorylated (H3Y41ph). Can interact with lamin-B receptor (LBR). This interaction can contribute to the association of the heterochromatin with the inner nuclear membrane. Involved in the formation of functional kinetochore through interaction with MIS12 complex proteins |
| 26 | CCDC93 | coiled-coil domain containing 93 |
| 27 | CCNJL | cyclin J-like |
| 28 | CCR1 | chemokine (C-C motif) receptor 1; Receptor for a C-C type chemokine. Binds to MIP-1-alpha, MIP-1-delta, RANTES, and MCP-3 and, less efficiently, to MIP-1- beta or MCP-1 and subsequently transduces a signal by increasing the intracellular calcium ions level. Responsible for affecting stem cell proliferation |
| 29 | CD44 | CD44 molecule (Indian blood group) |
| 30 | CDC42EP3 | CDC42 effector protein (Rho GTPase binding) 3; Probably involved in the organization of the actin cytoskeleton. May act downstream of CDC42 to induce actin filament assembly leading to cell shape changes. Induces pseudopodia formation in fibroblasts |
| 31 | CELF2 | CUGBP, Elav-like family member 2 |
| 32 | CEP350 | centrosomal protein 350kDa; Plays an essential role in centriole growth by stabilizing a procentriolar seed composed of at least, SASS6 and CENPJ. Required for anchoring microtubules to the centrosomes and for the integrity of the microtubule network. Recruits PPARA to discrete subcellular compartments and thereby modulates PPARA activity |
| 33 | CLCN6 | chloride channel, voltage-sensitive 6 |
| 34 | CLHC1 | clathrin heavy chain linker domain containing 1 |
| 35 | CLIP3 | CAP-GLY domain containing linker protein 3; Functions as a cytoplasmic linker protein. Involved in TGN-endosome dynamics |
| 36 | COX18 | cytochrome c oxidase assembly homolog 18 (yeast); Required for the insertion of integral membrane proteins into the mitochondrial inner membrane. Essential for the activity and assembly of cytochrome c oxidase. Plays a central role in the translocation and export of the C-terminal part of the COX2 protein into the mitochondrial intermembrane space |
| 37 | CPM | carboxypeptidase M; Specifically removes C-terminal basic residues (Arg or Lys) from peptides and proteins. It is believed to play important roles in the control of peptide hormone and growth factor activity at the cell surface, and in the membrane-localized degradation of extracellular proteins |
| 38 | CREBZF | CREB/ATF bZIP transcription factor; Strongly activates transcription when bound to HCFC1. Suppresses the expression of HSV proteins in cells infected with the virus in a HCFC1-dependent manner. Also suppresses the HCFC1- dependent transcriptional activation by CREB3 and reduces the amount of CREB3 in the cell. Able to down-regulate expression of some cellular genes in CREBZF-expressing cells |
| 39 | CRTAP | cartilage associated protein; Necessary for efficient 3-hydroxylation of fibrillar collagen prolyl residues |
| 40 | DBF4B | DBF4 homolog B (S. cerevisiae); Regulatory subunit for CDC7 which activates its kinase activity thereby playing a central role in DNA replication and cell proliferation. Required for progression of S and M phases. The complex CDC7-DBF4B selectively phosphorylates MCM2 subunit at 'Ser-40' and then is involved in regulating the initiation of DNA replication during cell cycle |
| 41 | DCTN4 | dynactin 4 (p62); Could have a dual role in dynein targeting and in ACTR1A/Arp1 subunit of dynactin pointed-end capping. Could be involved in ACTR1A pointed-end binding and in additional roles in linking dynein and dynactin to the cortical cytoskeleton |
| 42 | DCTN5 | dynactin 5 (p25) |
| 43 | DDX17 | DEAD (Asp-Glu-Ala-Asp) box helicase 17; RNA-dependent ATPase activity. Involved in transcriptional regulation. Transcriptional coactivator for estrogen receptor ESR1. Increases ESR1 AF-1 domain-mediated transactivation. Synergizes with DDX5 and SRA1 RNA to activate MYOD1 transcriptional activity and probably involved in skeletal muscle differentiation. Required for zinc-finger antiviral protein ZC3HAV1-mediated mRNA degradation |
| 44 | DIDO1 | death inducer-obliterator 1 |
| 45 | DLG2 | discs, large homolog 2 (Drosophila); Required for perception of chronic pain through NMDA receptor signaling. Regulates surface expression of NMDA receptors in dorsal horn neurons of the spinal cord. Interacts with the cytoplasmic tail of NMDA receptor subunits as well as inward rectifying potassium channels. Involved in regulation of synaptic stability at cholinergic synapses. Part of the postsynaptic protein scaffold of excitatory synapses (By similarity) |
| 46 | DSTYK | dual serine/threonine and tyrosine protein kinase |
| 47 | EAF1 | ELL associated factor 1; Acts as a transcriptional transactivator of ELL and ELL2 elongation activities |
| 48 | EBF1 | early B-cell factor 1; Transcriptional activator which recognizes variations of the palindromic sequence 5'-ATTCCCNNGGGAATT-3' (By similarity) |
| 49 | EFR3B | EFR3 homolog B (S. cerevisiae) |
| 50 | EID1 | EP300 interacting inhibitor of differentiation 1; Interacts with RB1 and EP300 and acts as a repressor of MYOD1 transactivation. Inhibits EP300 and CBP histone acetyltransferase activity. May be involved in coupling cell cycle exit to the transcriptional activation of genes required for cellular differentiation. May act as a candidate coinhibitory factor for NR0B2 that can be directly linked to transcription inhibitory mechanisms |
| 51 | ENSG00000224186 | HCG2045138; Uncharacterized protein |
| 52 | EPG5 | ectopic P-granules autophagy protein 5 homolog (C. elegans); Involved in autophagy. May play a role in a late step of autophagy, such as clearance of autophagosomal cargo |
| 53 | EXOSC3 | exosome component 3; Non-catalytic component of the RNA exosome complex which has 3'->5' exoribonuclease activity and participates in a multitude of cellular RNA processing and degradation events. In the nucleus, the RNA exosome complex is involved in proper maturation of stable RNA species such as rRNA, snRNA and snoRNA, in the elimination of RNA processing by-products and non-coding 'pervasive' transcripts, such as antisense RNA species and promoter-upstream transcripts (PROMPTs), and of mRNAs with processing defects, thereby limiting or excluding their export to the cytoplasm. The R [...] |
| 54 | FAM3C | family with sequence similarity 3, member C; May be involved in retinal laminar formation. Promotes epithelial to mesenchymal transition |
| 55 | FAM49A | family with sequence similarity 49, member A |
| 56 | FBXL17 | F-box and leucine-rich repeat protein 17; Substrate-recognition component of the SCF (SKP1-CUL1-F- box protein)-type E3 ubiquitin ligase complex (By similarity) |
| 57 | FCRLA | Fc receptor-like A |
| 58 | FMNL3 | formin-like 3; Plays a role in the regulation of cell morphology and cytoskeletal organization. Required in the control of cell shape and migration |
| 59 | GALNT10 | UDP-N-acetyl-alpha-D-galactosamine:polypeptide N-acetylgalactosaminyltransferase 10 (GalNAc-T10) |
| 60 | GINS4 | GINS complex subunit 4 (Sld5 homolog); The GINS complex plays an essential role in the initiation of DNA replication, and progression of DNA replication forks. GINS4 is important for GINS complex assembly. GINS complex seems to bind preferentially to single-stranded DNA |
| 61 | GLIS3 | GLIS family zinc finger 3; Acts as both a repressor and activator of transcription. Binds to the consensus sequence 5'-GACCACCCAC-3' (By similarity) |
| 62 | GPATCH4 | G patch domain containing 4 |
| 63 | GPATCH8 | G patch domain containing 8 |
| 64 | GPNMB | glycoprotein (transmembrane) nmb; Could be a melanogenic enzyme (By similarity) |
| 65 | GRINA | glutamate receptor, ionotropic, N-methyl D-aspartate-associated protein 1 (glutamate binding); Potential apoptotic regulator |
| 66 | GSK3B | glycogen synthase kinase 3 beta; Constitutively active protein kinase that acts as a negative regulator in the hormonal control of glucose homeostasis, Wnt signaling and regulation of transcription factors and microtubules, by phosphorylating and inactivating glycogen synthase (GYS1 or GYS2), EIF2B, CTNNB1/beta-catenin, APC, AXIN1, DPYSL2/CRMP2, JUN, NFATC1/NFATC, MAPT/TAU and MACF1. Requires primed phosphorylation of the majority of its substrates. In skeletal muscle, contributes to insulin regulation of glycogen synthesis by phosphorylating and inhibiting GYS1 activity and hence glyc [...] |
| 67 | GYPA | glycophorin A (MNS blood group); Glycophorin A is the major intrinsic membrane protein of the erythrocyte. The N-terminal glycosylated segment, which lies outside the erythrocyte membrane, has MN blood group receptors. Appears to be important for the function of SLC4A1 and is required for high activity of SLC4A1. May be involved in translocation of SLC4A1 to the plasma membrane. Is a receptor for influenza virus. Is a receptor for Plasmodium falciparum erythrocyte-binding antigen 175 (EBA-175); binding of EBA-175 is dependent on sialic acid residues of the O-linked glycans. Appears to [...] |
| 68 | HAUS3 | HAUS augmin-like complex, subunit 3; Contributes to mitotic spindle assembly, maintenance of centrosome integrity and completion of cytokinesis as part of the HAUS augmin-like complex |
| 69 | HMBOX1 | homeobox containing 1; Transcription factor. Isoform 1 acts as a transcriptional repressor. Isoform 4 has very low activity as a transcriptional repressor |
| 70 | HPCAL4 | hippocalcin like 4; May be involved in the calcium-dependent regulation of rhodopsin phosphorylation (By similarity) |
| 71 | HS2ST1 | heparan sulfate 2-O-sulfotransferase 1; Catalyzes the transfer of sulfate to the C2-position of selected hexuronic acid residues within the maturing heparan sulfate (HS). 2-O-sulfation within HS, particularly of iduronate residues, is essential for HS to participate in a variety of high- affinity ligand-binding interactions and signaling processes. Mediates 2-O-sulfation of both L-iduronyl and D-glucuronyl residues (By similarity) |
| 72 | IGF1 | insulin-like growth factor 1 (somatomedin C); The insulin-like growth factors, isolated from plasma, are structurally and functionally related to insulin but have a much higher growth-promoting activity. May be a physiological regulator of [1-14C]-2-deoxy-D-glucose (2DG) transport and glycogen synthesis in osteoblasts. Stimulates glucose transport in rat bone-derived osteoblastic (PyMS) cells and is effective at much lower concentrations than insulin, not only regarding glycogen and DNA synthesis but also with regard to enhancing glucose uptake |
| 73 | ITGA2 | integrin, alpha 2 (CD49B, alpha 2 subunit of VLA-2 receptor); Integrin alpha-2/beta-1 is a receptor for laminin, collagen, collagen C-propeptides, fibronectin and E-cadherin. It recognizes the proline-hydroxylated sequence G-F-P-G-E-R in collagen. It is responsible for adhesion of platelets and other cells to collagens, modulation of collagen and collagenase gene expression, force generation and organization of newly synthesized extracellular matrix |
| 74 | ITPRIP | inositol 1,4,5-trisphosphate receptor interacting protein; Enhances Ca(2+)-mediated inhibition of inositol 1,4,5- triphosphate receptor (ITPR) Ca(2+) release |
| 75 | IYD | iodotyrosine deiodinase |
| 76 | JPH3 | junctophilin 3; Junctophilins contribute to the formation of junctional membrane complexes (JMCs) which link the plasma membrane with the endoplasmic or sarcoplasmic reticulum in excitable cells. Provides a structural foundation for functional cross-talk between the cell surface and intracellular calcium release channels. JPH3 is brain- specific and appears to have an active role in certain neurons involved in motor coordination and memory |
| 77 | KANSL2 | KAT8 regulatory NSL complex subunit 2; As part of the NSL complex it is involved in acetylation of nucleosomal histone H4 on several lysine residues and therefore may be involved in the regulation of transcription |
| 78 | KAZN | kazrin, periplakin interacting protein |
| 79 | KCNJ10 | potassium inwardly-rectifying channel, subfamily J, member 10; May be responsible for potassium buffering action of glial cells in the brain. Inward rectifier potassium channels are characterized by a greater tendency to allow potassium to flow into the cell rather than out of it. Their voltage dependence is regulated by the concentration of extracellular potassium; as external potassium is raised, the voltage range of the channel opening shifts to more positive voltages. The inward rectification is mainly due to the blockage of outward current by internal magnesium. Can be blocked by [...] |
| 80 | KIF13A | kinesin family member 13A |
| 81 | MARVELD1 | MARVEL domain containing 1; Microtubule-associated protein that exhibits cell cycle- dependent localization and can inhibit cell proliferation and migration (By similarity) |
| 82 | MEF2C | myocyte enhancer factor 2C; Transcription activator which binds specifically to the MEF2 element present in the regulatory regions of many muscle- specific genes. Controls cardiac morphogenesis and myogenesis, and is also involved in vascular development. Plays an essential role in hippocampal-dependent learning and memory by suppressing the number of excitatory synapses and thus regulating basal and evoked synaptic transmission. Crucial for normal neuronal development, distribution, and electrical activity in the neocortex. Necessary for proper development of megakaryocytes and platel [...] |
| 83 | METTL20 | methyltransferase like 20; Probable methyltransferase (By similarity) |
| 84 | MGAT4A | mannosyl (alpha-1,3-)-glycoprotein beta-1,4-N-acetylglucosaminyltransferase, isozyme A; Glycosyltransferase that participates in the transfer of N-acetylglucosamine (GlcNAc) to the core mannose residues of N- linked glycans. Catalyzes the formation of the GlcNAcbeta1-4 branch on the GlcNAcbeta1-2Manalpha1-3 arm of the core structure of N-linked glycans. Essential for the production of tri- and tetra-antennary N-linked sugar chains. Involved in glucose transport by mediating SLC2A2/GLUT2 glycosylation, thereby controlling cell-surface expression of SLC2A2 in pancreatic beta cells |
| 85 | MIP | major intrinsic protein of lens fiber; Water channel. May be responsible for regulating the osmolarity of the lens. Interactions between homotetramers from adjoining membranes may stabilize cell junctions in the eye lens core (By similarity) |
| 86 | MKI67 | antigen identified by monoclonal antibody Ki-67; Thought to be required for maintaining cell proliferation |
| 87 | MMAA | methylmalonic aciduria (cobalamin deficiency) cblA type; Probable GTPase. May function as chaperone. May be involved in the transport of cobalamin (Cbl) into mitochondria for the final steps of adenosylcobalamin (AdoCbl) synthesis |
| 88 | MRPL11 | mitochondrial ribosomal protein L11 |
| 89 | MTUS2 | microtubule associated tumor suppressor candidate 2; Binds microtubules. Together with MAPRE1 may target the microtubule depolymerase KIF2C to the plus-end of microtubules. May regulate the dynamics of microtubules at their growing distal tip |
| 90 | MUM1L1 | melanoma associated antigen (mutated) 1-like 1 |
| 91 | MYO9A | myosin IXA |
| 92 | NAP1L5 | nucleosome assembly protein 1-like 5 |
| 93 | NCOA4 | nuclear receptor coactivator 4 |
| 94 | NPY1R | neuropeptide Y receptor Y1; Receptor for neuropeptide Y and peptide YY. The rank order of affinity of this receptor for pancreatic polypeptides is NPY > [Pro-34] PYY, PYY and [Leu-31, Pro-34] NPY > NPY (2-36) > [Ile-31, Gln-34] PP and PYY (3-36) > PP > NPY free acid |
| 95 | NUMB | numb homolog (Drosophila) |
| 96 | PACS1 | phosphofurin acidic cluster sorting protein 1 |
| 97 | PARVA | parvin, alpha; Plays a role in sarcomere organization and in smooth muscle cell contraction. Required for normal development of the embryonic cardiovascular system, and for normal septation of the heart outflow tract. Plays a role in sprouting angiogenesis and is required for normal adhesion of vascular smooth muscle cells to endothelial cells during blood vessel development (By similarity). Plays a role in the reorganization of the actin cytoskeleton, formation of lamellipodia and ciliogenesis. Plays a role in the establishement of cell polarity, cell adhesion, cell spreading, and dir [...] |
| 98 | PATE2 | prostate and testis expressed 2 |
| 99 | PCDHA1 | protocadherin alpha 1; Potential calcium-dependent cell-adhesion protein. May be involved in the establishment and maintenance of specific neuronal connections in the brain |
| 100 | PEG3 | paternally expressed 3 |
| 101 | PGLYRP4 | peptidoglycan recognition protein 4; Pattern receptor that binds to murein peptidoglycans (PGN) of Gram-positive bacteria. Has bactericidal activity towards Gram-positive bacteria. May kill Gram-positive bacteria by interfering with peptidoglycan biosynthesis. Binds also to Gram- negative bacteria, and has bacteriostatic activity towards Gram- negative bacteria. Plays a role in innate immunity |
| 102 | PHF21A | PHD finger protein 21A; Component of the BHC complex, a corepressor complex that represses transcription of neuron-specific genes in non-neuronal cells. The BHC complex is recruited at RE1/NRSE sites by REST and acts by deacetylating and demethylating specific sites on histones, thereby acting as a chromatin modifier. In the BHC complex, it may act as a scaffold. Inhibits KDM1A-mediated demethylation of 'Lys-4' of histone H3 in vitro, suggesting a role in demethylation regulation |
| 103 | PIK3R3 | phosphoinositide-3-kinase, regulatory subunit 3 (gamma); Binds to activated (phosphorylated) protein-tyrosine kinases through its SH2 domain and regulates their kinase activity. During insulin stimulation, it also binds to IRS-1 |
| 104 | PIP4K2B | phosphatidylinositol-5-phosphate 4-kinase, type II, beta; Participates in the biosynthesis of phosphatidylinositol 4,5-bisphosphate |
| 105 | PLEKHH1 | pleckstrin homology domain containing, family H (with MyTH4 domain) member 1 |
| 106 | POLR3B | polymerase (RNA) III (DNA directed) polypeptide B; DNA-dependent RNA polymerase catalyzes the transcription of DNA into RNA using the four ribonucleoside triphosphates as substrates. Second largest core component of RNA polymerase III which synthesizes small RNAs, such as 5S rRNA and tRNAs. Proposed to contribute to the polymerase catalytic activity and forms the polymerase active center together with the largest subunit. Pol III is composed of mobile elements and RPC2 is part of the core element with the central large cleft and probably a clamp element that moves to open and close the [...] |
| 107 | POM121 | POM121 transmembrane nucleoporin |
| 108 | POM121C | POM121 transmembrane nucleoporin C; Essential component of the nuclear pore complex (NPC). The repeat-containing domain may be involved in anchoring components of the pore complex to the pore membrane. When overexpressed in cells induces the formation of cytoplasmic annulate lamellae (AL) |
| 109 | PPAPDC2 | phosphatidic acid phosphatase type 2 domain containing 2; Phosphatase that dephosphorylates presqualene diphosphate (PSDP) into presqualene monophosphate (PSMP), suggesting that it may be indirectly involved in innate immunity. PSDP is a bioactive lipid that rapidly remodels to presqualene monophosphate PSMP upon cell activation. Displays diphosphate phosphatase activity with a substrate preference for PSDP > FDP > phosphatidic acid |
| 110 | PRELP | proline/arginine-rich end leucine-rich repeat protein; May anchor basement membranes to the underlying connective tissue (By similarity) |
| 111 | PROX1 | prospero homeobox 1; May play a fundamental role in early development of CNS. May regulate gene expression and development of postmitotic undifferentiated young neurons (By similarity) |
| 112 | PRX | periaxin; Seems to be required for maintenance of peripheral nerve myelin sheath. May have a role in axon-glial interactions, possibly by interacting with the cytoplasmic domains of integral membrane proteins such as myelin-associated glycoprotein in the periaxonal regions of the Schwann cell plasma membrane. May have a role in the early phases of myelin deposition |
| 113 | RAP2A | RAP2A, member of RAS oncogene family; Small GTP-binding protein which cycles between a GDP- bound inactive and a GTP-bound active form. In its active form interacts with and regulates several effectors including MAP4K4, MINK1 and TNIK. Part of a signaling complex composed of NEDD4, RAP2A and TNIK which regulates neuronal dendrite extension and arborization during development. More generally, it is part of several signaling cascades and may regulate cytoskeletal rearrangements, cell migration, cell adhesion and cell spreading |
| 114 | RAP2C | RAP2C, member of RAS oncogene family; Small GTP-binding protein which cycles between a GDP- bound inactive and a GTP-bound active form. May play a role in cytoskeletal rearrangements and regulate cell spreading through activation of the effector TNIK. May play a role in SRE-mediated gene transcription |
| 115 | RBM33 | RNA binding motif protein 33 |
| 116 | RGS7 | regulator of G-protein signaling 7; Inhibits signal transduction by increasing the GTPase activity of G protein alpha subunits thereby driving them into their inactive GDP-bound form. Activity on G(o)-alpha is specifically enhanced by the RGS6/GNG5 dimer. May play a role in synaptic vesicle exocytosis. May play important role in the rapid regulation of neuronal excitability and the cellular responses to short-lived stimulations (By similarity) |
| 117 | RHOU | ras homolog family member U; Acts upstream of PAK1 to regulate the actin cytoskeleton, adhesion turnover and increase cell migration. Stimulates quiescent cells to reenter the cell cycle. Has no detectable GTPase activity but its high intrinsic guanine nucleotide exchange activity suggests it is constitutively GTP- bound. Plays a role in the regulation of cell morphology and cytoskeletal organization. Required in the control of cell shape |
| 118 | RNF144A | ring finger protein 144A; E3 ubiquitin-protein ligase which accepts ubiquitin from E2 ubiquitin-conjugating enzymes UBE2L3 and UBE2L6 in the form of a thioester and then directly transfers the ubiquitin to targeted substrates (By similarity) |
| 119 | RNF39 | ring finger protein 39 |
| 120 | RPS6KA6 | ribosomal protein S6 kinase, 90kDa, polypeptide 6; Constitutively active serine/threonine-protein kinase that exhibits growth-factor-independent kinase activity and that may participate in p53/TP53-dependent cell growth arrest signaling and play an inhibitory role during embryogenesis |
| 121 | RPS6KC1 | ribosomal protein S6 kinase, 52kDa, polypeptide 1; May be involved in transmitting sphingosine-1 phosphate (SPP)-mediated signaling into the cell |
| 122 | SCN8A | sodium channel, voltage gated, type VIII, alpha subunit; Mediates the voltage-dependent sodium ion permeability of excitable membranes. Assuming opened or closed conformations in response to the voltage difference across the membrane, the protein forms a sodium-selective channel through which Na(+) ions may pass in accordance with their electrochemical gradient. In macrophages and melanoma cells, isoform 5 may participate in the control of podosome and invadopodia formation |
| 123 | SERPINA1 | serpin peptidase inhibitor, clade A (alpha-1 antiproteinase, antitrypsin), member 1 |
| 124 | SERTAD2 | SERTA domain containing 2; Acts at E2F-responsive promoters to integrate signals provided by PHD- and/or bromodomain-containing transcription factors (By similarity) |
| 125 | SLC1A2 | solute carrier family 1 (glial high affinity glutamate transporter), member 2; Transports L-glutamate and also L- and D-aspartate. Essential for terminating the postsynaptic action of glutamate by rapidly removing released glutamate from the synaptic cleft. Acts as a symport by cotransporting sodium |
| 126 | SLC30A4 | solute carrier family 30 (zinc transporter), member 4; Probably involved in zinc transport out of the cytoplasm, maybe by sequestration into an intracellular compartment |
| 127 | SLC41A1 | solute carrier family 41, member 1; Acts as a magnesium transporter that is responsive to magnesium balance |
| 128 | SOGA3 | SOGA family member 3 |
| 129 | SOX7 | SRY (sex determining region Y)-box 7; Binds to and activates the CDH5 promoter, hence plays a role in the transcriptional regulation of genes expressed in the hemogenic endothelium and blocks further differentiation into blood precursors (By similarity). May be required for the survival of both hematopoietic and endothelial precursors during specification (By similarity). Competes with GATA4 for binding and activation of the FGF3 promoter (By similarity). Represses Wnt/beta-catenin-stimulated transcription, probably by targeting CTNNB1 to proteasomal degradation. Binds the DNA sequence [...] |
| 130 | SPATA6L | spermatogenesis associated 6-like |
| 131 | STAMBP | STAM binding protein; Zinc metalloprotease that specifically cleaves 'Lys-63'- linked polyubiquitin chains. Does not cleave 'Lys-48'-linked polyubiquitin chains (By similarity). Functions at the endosome and is able to oppose the ubiquitin-dependent sorting of receptors to lysosomes. Plays a role in signal transduction for cell growth and MYC induction mediated by IL-2 and GM-CSF. Potentiates BMP (bone morphogenetic protein) signaling by antagonizing the inhibitory action of SMAD6 and SMAD7 |
| 132 | STARD13 | StAR-related lipid transfer (START) domain containing 13 |
| 133 | STK35 | serine/threonine kinase 35 |
| 134 | SYTL4 | synaptotagmin-like 4; Modulates exocytosis of dense-core granules and secretion of hormones in the pancreas and the pituitary. Interacts with vesicles containing negatively charged phospholipids in a Ca(2+)-independent manner (By similarity) |
| 135 | TDRD1 | tudor domain containing 1 |
| 136 | TFB1M | transcription factor B1, mitochondrial; S-adenosyl-L-methionine-dependent methyltransferase which specifically dimethylates mitochondrial 12S rRNA at the conserved stem loop. Also required for basal transcription of mitochondrial DNA, probably via its interaction with POLRMT and TFAM. Stimulates transcription independently of the methyltransferase activity |
| 137 | TGOLN2 | trans-golgi network protein 2 |
| 138 | TIPARP | TCDD-inducible poly(ADP-ribose) polymerase; Poly [ADP-ribose] polymerase using NAD(+) as a substrate to transfer ADP-ribose onto glutamic acid residues of a protein acceptor; repeated rounds of ADP-ribosylation leads to the formation of poly(ADPribose) chains on the protein, thereby altering the function of the target protein. May play a role in the adaptative response to chemical exposure (TCDD) and thereby mediates certain effects of the chemicals (By similarity) |
| 139 | TMEM106C | transmembrane protein 106C |
| 140 | TMEM14E | transmembrane protein 14E |
| 141 | TPCN2 | two pore segment channel 2; Nicotinic acid adenine dinucleotide phosphate (NAADP) receptor that may function as one of the major voltage-gated Ca(2+) channels (VDCC) across the lysosomal membrane. May be involved in smooth muscle contraction |
| 142 | TRIM62 | tripartite motif containing 62 |
| 143 | TRUB1 | TruB pseudouridine (psi) synthase homolog 1 (E. coli); May be responsible for synthesis of pseudouridine from uracil in transfer RNAs (By similarity) |
| 144 | TTC26 | tetratricopeptide repeat domain 26; May play a critical role in ciliogenesis and normal cilia function (By similarity) |
| 145 | UBXN4 | UBX domain protein 4; Involved in endoplasmic reticulum-associated protein degradation (ERAD) |
| 146 | UNC5B | unc-5 homolog B (C. elegans); Receptor for netrin required for axon guidance. Mediates axon repulsion of neuronal growth cones in the developing nervous system upon ligand binding. Axon repulsion in growth cones may be caused by its association with DCC that may trigger signaling for repulsion. It also acts as a dependence receptor required for apoptosis induction when not associated with netrin ligand. Mediates apoptosis by activating DAPK1. In the absence of NTN1, activates DAPK1 by reducing its autoinhibitory phosphorylation at Ser-308 thereby increasing its catalytic activity |
| 147 | ZBTB7A | zinc finger and BTB domain containing 7A; Plays a key role in the instruction of early lymphoid progenitors to develop into B lineage by repressing T-cell instructive Notch signals (By similarity). Specifically represses the transcription of the CDKN2A gene. Efficiently abrogates E2F1- dependent CDKN2A transactivation/de-repression. Binds to the consensus sequence 5'-[GA][CA]GACCCCCCCCC-3' (By similarity) |
| 148 | ZDHHC3 | zinc finger, DHHC-type containing 3; Palmitoyltransferase with broad specificity. Palmitoylates GABA receptors on their gamma subunit (GABRG1, GABRG2 and GABRG3), which regulates synaptic clustering and/or cell surface stability. Palmitoylates glutamate receptors GRIA1 and GRIA2, which leads to their retention in Golgi (By similarity) |
| 149 | ZNF169 | zinc finger protein 169; May be involved in transcriptional regulation |
| 150 | ZNF37A | zinc finger protein 37A; May be involved in transcriptional regulation |
| 151 | ZNF540 | zinc finger protein 540; May act as a transcriptional repressor |
| 152 | ZNF609 | zinc finger protein 609 |
